# Combination effect of voluntary exercise and garlic (*Allium sativum*) on oxidative stress, cholesterol level and histopathology of heart tissue in type 1 diabetic rats

**DOI:** 10.15171/jcvtr.2019.10

**Published:** 2019-03-06

**Authors:** Rafighe Ghyasi, Gisou Mohaddes, Roya Naderi

**Affiliations:** ^1^Drug Applied Research Center of Tabriz University of Medical Sciences, Tabriz, Iran; ^2^Neuroscience Research Centre of Tabriz University of Medical Sciences, Tabriz, Iran; ^3^Nephrology and Kidney Transplant Research Center, Urmia University of Medical Sciences, Urmia, Iran; ^4^Department of Physiology, Faculty of Medicine, Urmia University of Medical Sciences, Urmia, Iran

**Keywords:** Voluntary Exercise, Garlic, MDA, TAC, Cholesterol, Oxidative Stress

## Abstract

***Introduction:*** This study aimed to evaluate the combination effect of voluntary exercise and garlic on oxidative markers, cholesterol level and histological alterations in streptozotocin (STZ)- induced diabetes in rat heart.

***Methods:*** Thirty-five male Wistar rats were randomly assigned into five experimental groups (n=7): Control, Diabetes, Diabetes+Garlic, Diabetes+Exercise, Diabetes+Garlic+Exercise groups. Diabetes was induced by STZ (ip, 50 mg/kg) in animals. Animals received garlic homogenate (250 mg/kg) by oral gavage or subjected to voluntary exercise alone or together for 6 weeks. At the end of intervention blood and heart tissue samples were obtained and used for measurement of glycosylated haemoglobin (HbA1c), cholesterol, total antioxidant capacity (TAC), malondialdehyde (MDA) levels and histological analysis.

***Results:*** Improved blood glucose, cholesterol, total antioxidant capacity, and MDA levels were established in both Diabetes+Garlic and Diabetes+Exercise groups. Additionally, staining with Hematoxylin and Eosin (H&E) revealed that voluntary exercise and garlic alone and together reduced interstitial edema, leukocyte infiltration, and myonecrosis. Furthermore, simultaneous treatment of diabetic rats with garlic and exercise training had an additive effect on these parameters.

***Conclusion:*** The findings indicated that combination therapy with garlic and voluntary exercise may present more beneficial effects in heart histological remodeling in diabetic rats than the use of garlic or voluntary exercise alone and that these beneficial effects might be associated with enhancement in cholesterol, total antioxidant capacity, and MDA levels.

## Introduction

Diabetes mellitus (DM) is associated with an increased risk of cardiovascular disease.^1^ The prevalence of vascular disease in diabetic patients is rised more than 3-fold and is the crucial cause of mortality and morbidity in diabetic patients.^2^ It has been reported that prolonged hyperglycemia leads to generation of free radicals, oxidative stress, and inflammation.^3^ These free radicals play in various physiological events including gene expression, signaling pathways and cellular defense against pathogens.^4^ In pathological conditions, such as uncontrolled hyperglycemia, free radicals are assembled in order to the activation of several pathways.^5^ Hyperglycemia stimulates protein kinase C (PKC) and poly-ADP ribose polymerase (PARP) pathways; as well as it increases production of advanced glycation outcomes, polyol and hexosamine pathways. These pathways are stimulated or increased in order to mitochondrial superoxide overproduction.^6^ Furthermore, free radical overload and formation of oxidative stress lead to antioxidant defense system attenuation in tissues during diabetes.^7,8^ Diabetes-induced oxidative damage in different organs including the cardiovascular system, as manifested by increased MDA and also decreased antioxidant enzymes activities.^9^ Exercise training has been reported to reverse oxidative stress and provide alleviating effects on hyperglycemia and hyperlipidemia in the cardiomyocytes.^10^ Recently, Dietary plants, identified from herbal medicine are providing an opportunity for the apperance of new types of therapeutics.^11^ Among them, garlic (*Allium sativum*) has been utilized in herbal medicine for its unique antioxidant and anti-inflammatory properties.^11^ Garlic may markedly modulate antioxidants status in the blood and heart of type 1diabetic rats.^6^ Although the combination effect of voluntary exercise and garlic in diabetic heart related to oxidative stress and lipid parameters have not been elucidated yet. According to above, in this study, we decided to evaluate the combination effect of garlic and voluntary exercise performance on histological parameters, cholesterol level and oxidative markers in the blood and heart of type 1 diabetic rats.

## Materials and Methods

### Animals and experimental design

Male Wistar rats (200-250 g) were housed under controlled environmental conditions and maintained on a 12h light-dark cycle in an air-conditioned room and stable temperature (22±1℃) room with food and water provided ad libitum. Thirty-five animals were randomly assigned to five experimental groups (n = 7).^6^

Control: Animals that received 0.4 mL of sodium citrate buffer, pH 4.5.

Diabetes: Animals that received a single dosage of 50 mg/kg of STZ intraperitoneally (i.p).

Diabetes + Garlic: STZ (50 mg/kg, i.p) was administered to animals by a single injection. After confirmation of diabetes, 250 mg/kg of homogenized garlic was fed by oral gavage 6 days a week for a period of 6 weeks.^6,12^

Diabetes+Exercise: STZ (50 mg/kg, i.p) was administered to animals by a single injection. After confirmation of diabetes, voluntary running wheel exercise was performed for the animals for 6 weeks.

Diabetes+Garlic+Exercise: Diabetic rats that received garlic homogenate (250 mg/kg ) 6 days a week by gavage and performed voluntary exercise simultaneously for 6 weeks.

Diabetes was induced with a single injection of STZ (50 mg/kg) (Sigma Chemical Co., St Louis, MO, USA) intraperitoneally. STZ dissolved in 0.4 mL of sodium citrate buffer, pH 4.5. To confirm the induction of diabetes, 72 hours later after the STZ injection, blood glucose level was measured by a glucometer (Elegance, CT-X10., Frankenberg, Germany). The fasting glucose concentration of more than 300 mg/dL was considered diabetic.^12^

### Preparing Garlic Homogenate

Garlic (*Allium sativum*) bulbs were obtained from a local market. Cloves were peeled, cut off, blend by a mixing machine and form a paste, afterwards, suspended in distilled water. The garlic homogenate was provided freshly each day.^12^

### Voluntary exercise

The animals in the voluntary exercise groups were housed in a separate cages with stainless-steel running wheels (1.00 m circumference, TajhizGostar) and were subjected free access to the wheel 24 hours per day for 6 weeks. The wheels were attached to a permanent sensor for counting of revolutions. Running distance was monitored daily. If the running distance was less than 2000 m/d, that animal was excluded from the study. Control rats were kept in standard holding cages without running wheels during 6 weeks.^13^

### Tissue processing and homogenate preparation

At the end of experiment, the animals were deeply anesthetized with pentobarbital sodium (Sigma-Aldrich; St. Louis, Missouri, United States) (35 mg/kg, i.p.). Then the thoracic cavity was disclosed and blood samples were obtained by syringes from the heart of anesthetized rats. Some blood centrifuged at 4000 ×g for 20 minutes immediately. The serum obtained and stored in tubes at −80°C for determination of cholesterol, TAC and MDA levels. Some blood as whole blood used for measuring HbA1c (glycosylated haemoglobin) level. Hearts were isolated, frozen in liquid nitrogen and stored at deep freeze (-70°C). For MDA estimation heart samples were homogenized in 1.15% KCl solution (Sigma-Aldrich; St. Louis, Missouri, United States). The homogenates were centrifuged at 1000 rpm for 1 minute at 4°C, and then stored at −80°C for later measurements.^13,14^

### MDA and TAC assessment

MDA as the end-product of lipid peroxidation was evaluated in the blood and tissue samples as described by the Esterbauer and Cheeseman method. Accordingly, MDA reacts with thiobarbituric acid (Sigma-Aldrich; St. Louis, Missouri, United States), and the pink pigment was produced, that has a maximum absorption at 532 nm.^13,14^

TAC was assessed by a Randox (Crumlin, County Antrim, United Kingdom) total antioxidant kit. Accordingly, 2,2’-azino-bis(3-ethylbenzothiazoline-6-sulfanate) (ABTS) is incubated with peroxidase and H2O2 resulting in the radical cation ABTS ^+^ production. Finally, at 600 nm a stable blue-green color was observed.^13^

### Detection of fasting blood glucose, HbA1c and serum total cholesterol

Blood samples were obtained from the tip of tail and glucose levels were assesed by a digital glucometer (Elegance, CT-X10., Frankenberg, Germany).

HbA1c was also evaluated in whole blood by immunoturbidimetry kit (Pars Azmoon HbA1c kit, Iran) according to manufacturer´s structure. Serum samples were evaluated for total cholesterol levels using an auto blood analyzer (Bayer Corp. USA). The related kit was purchased from Pars Azmoon CO, Iran.

### Histological evaluation

To perform histopathological staining, heart tissues were immediately excised and fixed in 10% buffered-formalin solution. After dehydration in ascending grades of alcohol, tissues were embedded in paraffin. To evaluate interstitial edema, congestion, leukocytosis infiltration, and myonecrosis, H&E staining was applied. Sections of 5 μm were obtained, stained and evaluated under a light microscope (Olympus BH-2, Tokyo, Japan) by a blinded pathologist. Histological changes were scored as follows: (-) none, (+) mild, (++) moderate, and (+++) severe damage.^15^

### Statistical analysis

Evaluating of Normality was checked by Kolmogorov-Smirnov test. Data were statistically evaluated by one-way analysis of variance (ANOVA) followed by Tukey’s test. The significant level was considered at *P *< 0.05. Data are expressed as means ± SEM.

## Results

### Lipid peroxidation and total antioxidant capacity

Table 1 shows that a significant increase in MDA level (*P *< 0.001) in the heart and serum of the diabetic group compared with the control group. However, 6 weeks treatment of the diabetic animals with garlic or exercise alone (*P *< 0.01) and in combination (*P *< 0.001) significantly decreased serum MDA level in comparison to the group with diabetes. Also garlic (*P *< 0.01) and exercise (*P *< 0.001) treatment and their combination (*P *< 0.001) in the diabetic heart reversed MDA level. Furthermore, in the diabetic animals, combination therapy with garlic and exercise had a signiﬁcant reduction in heart MDA compared to groups treated with garlic (*P *< 0.01) or exercise (*P *< 0.05) alone. In addition, we found that in the groups with garlic administration or exercise performance alone (*P *< 0.01) or together (*P *< 0.001) serum TAC level appeared to be signiﬁcantly higher than that in the diabetic group. Also, combination therapy of rats with garlic and exercise had a higher TAC level (*P *< 0.05) in the serum of diabetic rats compared with Diabetes+Exercise group.

**Table 1:** Effect of garlic and voluntary exercise and their combination of changes to heart tissue

**Parameters**	**Control**	**Diabetes**	**Diabetes + Garlic**	**Diabetes+ Exercise**	**Diabetes + Garlic +Exercise**
TAC (mmol/L)	0.81±0.031	0.54±0.05^a^	1.01±0.09^c^	0.91±0.07^c^	1.25±0.11^d,e^
Heart MDA (nmol/mgpr)	7.12±0.55	13.6±0.74^b^	9.43±0.79^c^	8.54±0.63^d^	5.35±0.31^d,e,f^
Serum MDA (nmol/mL)	3.2±0.66	7.8±0.95^b^	3.9±0.27^c^	3.9±0.23^c^	2.9±0.18^d^

^a^ *P* < 0.05, ^b^*P* < 0.001 versus Control group. ^c^*P* < 0.01, ^d^*P* < 0.001 versus Diabetes group. ^e^*P* < 0.05 versus Diabetes + Exercise group. ^f^*P* < 0.01 versus Diabetes + Garlic group. Total antioxidant capacity (TAC), heart and serum malondialdehyde (MDA) levels in STZ-induced diabetic rats after 6 weeks. Data are shown as means ± SEM.

### Detection of fasting blood glucose, HbA1c and serum total cholesterol

**Figure 1:**
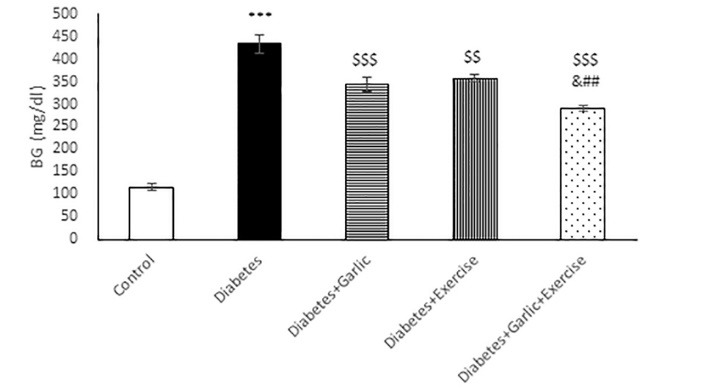
Effect of garlic and voluntary exercise on blood glucose (BG) at the end of experiment. Data are represented as the mean ± SEM (n = 7). *** *P*< 0.001 versus Control group. $$ *P* < 0.01, $$$ *P* < 0.001 versus Diabetes group. ## *P* < 0.01 versus Diabetes + Exercise group. & *P* < 0.05 versus Diabetes + Garlic group.

**Figure 2:**
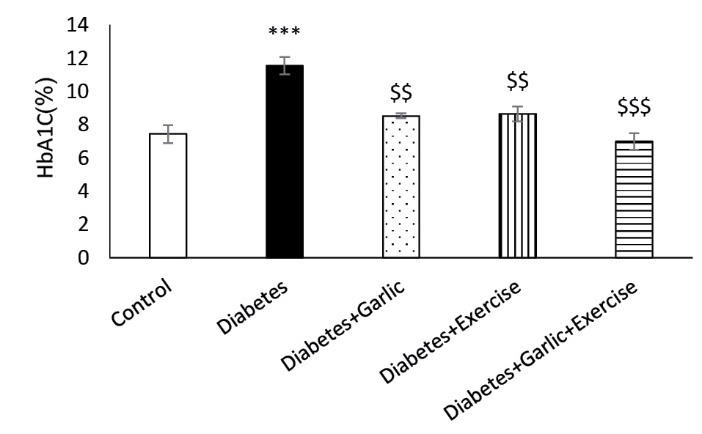
Effect of garlic and voluntary exercise on HbA1C (Glycosylated Hemoglobin) at the end of experiment. Data are represented as the mean ± SEM (n = 7). *** *P* < 0.001 versus Control group. $$ *P* < 0.01, $$$ *P* < 0.001 versus Diabetes group.

**Figure 3:**
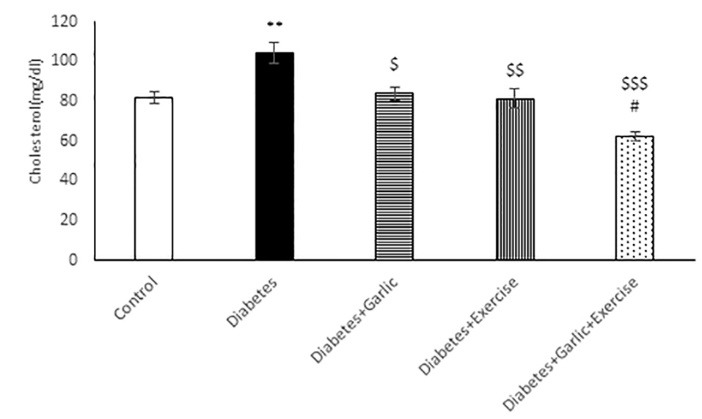
Effect of garlic and voluntary exercise on cholesterol at the end of experiment. Data are represented as the mean ± SEM (n = 7). ** *P* < 0.001 versus Control group. $ *P* < 0.05, $$ *P* < 0.01, $$$ *P* < 0.001 versus Diabetes group. # *P* < 0.05 versus Diabetes+Exercise group.

As shown in Figure 1, after 6 weeks of treatment, blood glucose level was significantly lower in Diabetes + Garlic (*P *< 0.001), Diabetes+Exercise (*P *< 0.01), and Diabetes+Garlic+Exercise (*P *< 0.001) groups than the diabetic group. Furthermore, simultaneous treatment of the diabetic group with garlic and exercise significantly decreased blood glucose level (*P *< 0.001) compared to the Diabetes+Garlic (*P *< 0.05) and Diabetes+Exercise (*P *< 0.01) groups. HbA1c level was also evaluated in all groups; it was significantly (*P *< 0.001) higher in diabetic rats compared to the control rats (Figure 2). Garlic administration or exercise training alone (*P *< 0.01) or in combination (*P *< 0.001) also significantly decreased HbA1C level in diabetic rats compared to the diabetes group. Total cholesterol level in the serum of diabetic group was significantly (*P *< 0.01) higher than in the control rats. Garlic (*P *< 0.05) and exercise training (*P *< 0.01) alone and together (*P *< 0.001) significantly reversed the increase in total cholesterol level in the serum of diabetic rats. Furthermore, simultaneous treatment with garlic and exercise in diabetic group significantly (*P *< 0.05) decreased cholesterol level compared to Diabetes+Exercise group (Figure 3).

### Histopathological findings

**Figure 4:**
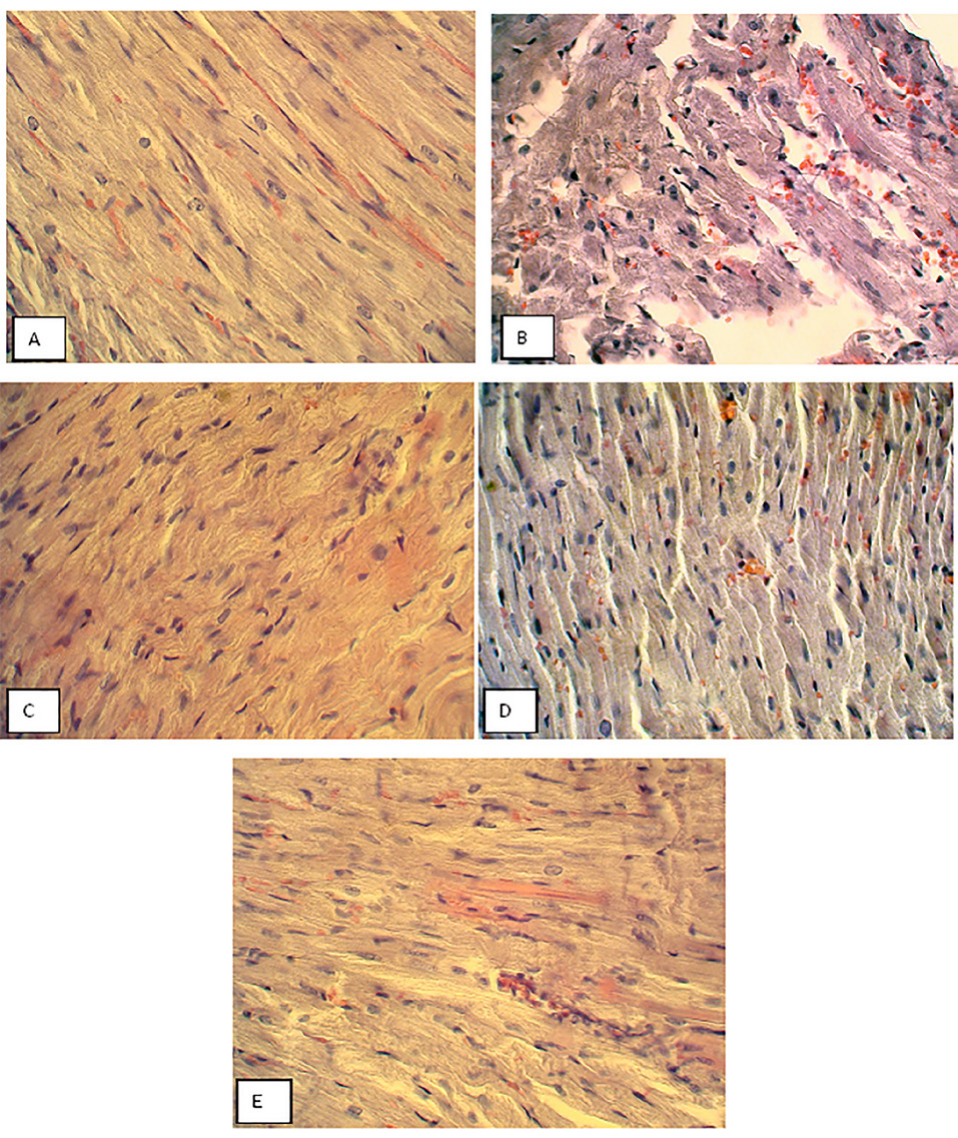
Histopathological evaluation of rat cardiomyocytes in different groups at the end of the experiment. Heart sections were stained by hematoxylin and eosin (H&E) and examined by a light microscope (40 × HE).

Hematoxylin and Eosin staining was done to assess the effect of garlic and voluntary exercise on heart tissue alterations in diabetic animals. According to Figure 4, there is a normal structure with heart tissue in the control group (Figure 4A). In the diabetic group, histological alterations including interstitial edema, leukocyte infiltration, as well as, myonecrosis were higher than those of the control animals (Figure 4B). Garlic consumption and voluntary exercise performance attenuated all histological changes in the heart tissue of the treated groups compared to the diabetic group (Figure 4C, D). Furthermore, simultaneous treatment with garlic and exercise resulted in a marked attenuation of interstitial edema, leukocyte infiltration, and myonecrosis in diabetic animals (Figure 4E, Table 2).

**Table 2:** Comparison of histological changes of cardiomyocytes in different groups of rats (hematoxylin and eosin)

	**Interstitial edema and congestion**	**Leukocyte infiltration**	**Cardiomyocyte necrosis**
Control	-	-	-
Diabetes	+++	+++	+++
Diabetes+Garlic	++	++	++
Diabetes+Exercise	++	++	++
Diabetes+Garlic+Exercise	+	+	+

A minimum of 10 fields for each cardiomyocyte slide was evaluated and assigned for severity of changes using scores on a scale of (–) none, (+) mild, (++) moderate, and (+++) severe damage (n =7 for each group).

## Discussion

The results of the present study indicated that oral administration of garlic in combination with voluntary exercise significantly reduced histological alterations in the heart of diabetic rats. These alterations probably were mediated by modification in oxidative and lipid markers. We observed the significant elevation in TAC level with a reduction in MDA and cholesterol contents in both diabetes+garlic and diabetes+ exercise groups compared with the diabetic group. Moreover, our data demonstrated the favorable and additive effects of garlic in combination with voluntary exercise on histological alterations, reducing oxidative stress and cholesterol level in diabetic rats.

Diabetes mellitus is a serious chronic metabolic disease which is associated with hyperglycemia and several complications including cardiovascular disease.^1^ In this study, the increased level of blood glucose and HbA1c in STZ-induced diabetic rats were reduced by oral administration of garlic alone and in combination with voluntary exercise. Our findings match with the studies that reported the antidiabetic and cardioprotective activity of garlic and voluntary training alone in a diabetic situation.^16,17^ Furthermore, as we reported, combined intervention is more effective in reducing blood glucose and HbA1c levels than either intervention alone. It is obvious that HbA1c demonstrates the percentage of hemoglobin bound to glucose. It is a very sensitive index for glycemic control. It has been reported that the reduction of HbA1c level resulted in the lower risk of cardiovascular complications.^18^

The pathogenesis of diabetic cardiomyopathy which is a multifactorial process, includes metabolic disturbances including increased oxidative stress and modulated non-oxidative glucose pathways and lipid metabolism.^19^ Lipid peroxidation is a key marker of oxidative stress which causes tissue damage in a diabetic situation, especially in association with atherosclerosis and cardiovascular disease.^18,20^ Indeed, free radicals are known to be associated with the formation of oxidized LDL which stimulates the release of ROS and in turn promotes atherosclerosis.^21^Since diabetes-induced oxidative damage has a pivotal role in heart malfunction including cardiomyopathy, it has been suggested that restoring redox balance in heart tissue can combat oxidative damage and alleviate diabetic complications.^22,23^ So, we evaluated the combination eﬀect of garlic and voluntary exercise as potent stimuli for the endogenous antioxidant system. In our study, garlic and voluntary exercise significantly increased antioxidant defense system potency by improving TAC activity and decreasing MDA production in cardiomyocytes. Interestingly, aside from improved oxidative stress, in this study, a parallel decrease in cholesterol level was seen following garlic and voluntary exercise in diabetic animals. This finding is the ﬁrst to demonstrate antioxidative activity and cholesterol-lowering effect of garlic and voluntary exercise together in an additive manner in the heart tissue of diabetic animals. In addition, histopathology of cardiomyocytes was also performed to corroborate the findings of the biochemical investigation. According to previous studies, exercise is a modulating factor which can provide several useful effects on diabetes complication including cardiovascular disorders.^9,24^ It has shown that moderate-intensity physical activity improves myocardial dysfunction of STZ induced diabetes.^9,25^ Also, exercise training is a beneficial non-pharmacological therapy which can modulate oxidative stress in diabetes and improve antioxidant defenses system, mitochondrial function and physiological cardiac growth.^26^ Exhaustive physical activity can contribute to inflammation and oxidative stress.^27^ However, it was reported that voluntary exercise as a non-exhaustive exercise has beneficial effects on activation of the certain antioxidant defense system and substantially an increase in TAC.^28^ Furthermore, previous studies showed that exercise improved lipid profiles of healthy,^29,30^ and diabetic rats.^31,32^ The molecular mechanisms by which voluntary exercise provides a protective effect on the diabetic heart are not fully elucidated. However, accumulating evidence clarified that the redox state improvement and the increases of generation and bioavailability of NO may be involved in this process.^33^ In addition, upregulation of taurine in exercise training could attenuate reactive oxygen species production. Taurine may be a mechanism that reduces lipid peroxides and also restores the level of SOD in the diabetic heart.^34^ In addition, upregulation of triglyceride lipolysis, improvement of antioxidant/oxidant ratio, and modified synthesis of LDL-C or elimination rate of LDL-C from the plasma suggested for the lipid-lowering effect of exercise training in STZ induced diabetic rats.^29,35,36^ Moreover, multiple lines of evidence highlighted that garlic had a variety of medicinal properties including hypoglycaemic, hypocholesterolemic and hypolipidaemic activities. Also, in our previous studies, we have obtained consistent results using homogenized garlic in diabetic heart and explaining the protective effect of this popular herb in preventing oxidative stress.^6^ Due to the beneficial effects of garlic and voluntary exercise mentioned above, in this study, we decided to explore the combination effect of garlic with voluntary exercise on oxidative and lipid markers in diabetic animals. According to our data, simultaneous treatment with garlic and voluntary exercise lead to a higher increase of TAC level and a more reduced MDA content and cholesterol level than either of them alone in the serum and heart of type 1 diabetic rats. It was claimed that garlic improves SOD, GPX, and CAT activities.^6^ This effect could be contributed primarily to a variety of sulfur-containing compounds and their precursors.^37^ Furthermore, bioactive components of garlic may probably chelate the metal ions (Cu, Zn, Mn) and result in scavenge the superoxide ions leading to cellular redox status balance.^38^ In addition, it was indicated that oxidative stress caused by the production of free radicals exacerbated by hypercholesterolemia may lead to endothelial dysfunction.^39^ Therefore we also measured cholesterol level in the serum of diabetic animals following garlic consumption and voluntary exercise performance. In general, blood lipid levels have an important role in preventing long-term complications of DM including atherosclerosis.^40^ The mechanism of lipid-lowering effect of garlic in part is inhibition of lipogenesis and stimulation of lipolysis in mature 3T3-L1 adipocytes.^41^ However, molecular mechanisms for the protective effect of combined garlic and voluntary exercise during diabetes is unclear and requires further investigation.

## Conclusion

In conclusion, this study demonstrated that garlic and voluntary exercise alone or together reduced blood glucose, HbA1c, cholesterol, lipid peroxidation marker and improved antioxidant defense system in rats with type 1 diabetes. In addition, combined treatment of garlic and voluntary exercise is more effective in controlling above mentioned parameters in diabetic animals. These findings briefly reported the benefits of combination effect of garlic and voluntary exercise on cholesterol, redox state, and glycemic parameters. Therefore, we confirmed the potential value of combination therapy in diabetic individuals.

## Ethical approval

This study was agreed by the Ethics Committee on Animal Experiments of the Tabriz University of Medical Sciences under the protocol number 91.4-2.4.

## Competing interests

All authors declare no competing financial interests exist.

## Acknowledgements

This study was supported by a grant from the Drug Applied Research Centre, Tabriz University of Medical Sciences, Tabriz, Iran.
